# Effect of Constrained Arm Posture on the Processing of Action Verbs

**DOI:** 10.3389/fnins.2017.00057

**Published:** 2017-02-10

**Authors:** Masaaki Yasuda, John F. Stins, Takahiro Higuchi

**Affiliations:** ^1^Department of Health Promotion Science, Tokyo Metropolitan UniversityTokyo, Japan; ^2^Faculty of Behavioural and Movement Sciences, Department of Human Movement Sciences, MOVE Research Institute Amsterdam, Vrije Universiteit AmsterdamAmsterdam, Netherlands

**Keywords:** embodied language, cognition, peripheral bodily state, language, action-related verb

## Abstract

Evidence is increasing that brain areas that are responsible for action planning and execution are activated during the information processing of action-related verbs (e.g., *pick* or *kick*). To obtain further evidence, we conducted three experiments to see if constraining arm posture, which could disturb the motor planning and imagery for that arm, would lead to delayed judgment of verbs referring to arm actions. In all experiments, native Japanese speakers judged as quickly as possible whether the presented object and the verb would be compatible (e.g., *ball–throw*) or not (e.g., *ball–pour*). Constrained arm posture was introduced to the task by asking participants to keep both hands behind their back. Two types of verbs were used: manual action verbs (i.e., verbs referring to actions performed on an object by a human hand) and non-manual action verbs. In contrast to our hypothesis that constrained arm posture would affect only the information processing of manual action verbs, the results showed delayed processing of both manual action and non-manual action verbs when the arm posture was constrained. The effect of constrained arm posture was observed even when participants responded with their voice, suggesting that the delayed judgment was not simply due to the difficulty of responding with the hand (i.e., basic motor interference). We discussed why, contrary to our hypothesis, constrained arm posture resulted in delayed CRTs regardless of the “manipulability” as symbolized by the verbs.

## Introduction

Several lines of evidence show that brain regions that are involved in motor planning and execution are also involved in the semantic processing of language stimuli (Aziz-Zadeh and Damasio, 2008; Pulvermüller and Fadiga, 2010; Willems and Casasanto, 2011). The first line of evidence comes from brain imaging and neurophysiological studies. Brain areas that are activated while performing finger movements are also activated when individuals passively read words regarding arm actions (Hauk et al., 2004). Premotor areas, which play a role in planning movements and in the sensory guidance of movements, are activated during the semantic processing of action-related words (Hauk et al., 2004; Shtyrov et al., 2004; Pulvermüller et al., 2005; Tettamanti et al., 2005; Aziz-Zadeh et al., 2006; Raposo et al., 2009; Willems et al., 2011; Michael et al., 2014). Buccino et al. (2005) used transcranial magnetic stimulation to assess whether listening to action-related sentences modulates the activity of the primary motor cortex. The results showed that motor activity in the primary motor cortex was elicited soon after the presentation of action-related sentences and that stimulation of that region at early latencies interfered with language task performance. These results hint at the involvement of brain areas responsible for action planning and execution in information processing for action-related words.

Behavioral studies showed evidence along the same lines (Glenberg and Kaschak, 2002; Boulenger et al., 2008, 2009; Dalla Volta et al., 2009; Aravena et al., 2010; Rueschemeyer et al., 2010; Springer and Prinz, 2010; Costantini et al., 2011; Ambrosini et al., 2012; Liepelt et al., 2012; van Dam et al., 2014). For example, in one study (Glenberg and Kaschak, 2002), participants were asked to judge whether a sentence was semantically meaningful. The response involved moving the index finger from the resting position to either a button located away from the trunk or a button located closer to the trunk. The results showed that, when a sentence implied manual action whereby the hand would move away from the trunk (e.g., “Put your finger under the faucet”), participants' responses were delayed when the response involved moving the finger closer to the trunk. The authors reasoned that action-related sentences referring to movement in a particular direction are processed by brain areas that are also responsible for planning and executing that action. As a result, movement execution in the opposite direction was delayed. In another study (Ambrosini et al., 2012), participants were asked to judge as quickly as possible whether a presented object and a verb were compatible, meaning that the action symbolized by the verb could be sensibly carried out on the object. If the object and the verb were judged to be compatible, participants were asked to lift the right index finger from a response button and then mimic a reach-to-grasp movement toward the computer screen. The results showed that judging the compatibility between object and verb was faster when the verb was related to the actual manipulation of that object than when the verb was related to simply observing the object (e.g., *to look*), and when the verb was related to the pointing toward the object (e.g., *to point*). These results suggest that there is likely an overlap between systems involved in information processing of action-related verbs, and perceptual-motor systems that are recruited during performance of that same action.

Neuropsychological studies showed that selective deficits exist in the processing of action-related words following lesions in motor regions of the brain (Bak et al., 2001; Neininger and Pulvermüller, 2003; Mahon and Caramazza, 2005). Neininger and Pulvermüller (2003) demonstrated that patients with lesions in the right frontal lobe (including primary motor and premotor areas) showed severe deficits in processing action verbs. Most patients had left hemiparesis. The experimental task was a lexical decision task in which participants determined as quickly as possible whether a presented letter string was a real word or a meaningless pseudoword. The words presented were either concrete nouns with strong visual associations (e.g., *cat*), concrete nouns with strong visual and motor associations (e.g., *train*), or action verbs that caused strong motor associations (e.g., *write*). The results showed that patients had more errors for action verbs than for other types of words. This suggests that motor dysfunction patients were also impaired in action-verb language processing, even in the absence of specific speech disorders.

The above studies show that neural systems subserving language and neural systems subserving motor control are strongly coupled. There is evidence in the field of mental imagery that when the arm is temporally prevented from moving, this can have a negative effect on the ability to perform various imagery tasks. For example, short-term (24 h) limb immobilization led to reduced performance in mental rotation (Meugnot et al., 2014). In another study, the duration of the motor imagery of hand movement was prolonged during load attachment to the arm (Cerritelli et al., 2000). Following the same logic, we reasoned that restriction of arm movements could lead to delayed processing of verbal material relating to manual activities. To our knowledge, this straightforward behavioral intervention has never been used in the field of language processing and might thus be used as a heuristic to further investigate whether changes in action capabilities have an impact on cognition.

A relevant study showed that patients who had suffered spinal cord inflammation and resultant neurological deficits (peripheral or musculoskeletal system impairment) had preserved information processing abilities regarding action-related words (Cardona et al., 2014). Although these findings seem to be inconsistent with our expectation, the major issue of the patients was not peripheral body status but impairment of the central nervous system Therefore, testing the effect of constraining limbs in individuals with no neurological or peripheral deficits is necessary to directly test this issue. Note that patients with neurological deficits are characterized by prolonged and profound changes in action capabilities, which tend to lead to neural reorganization (either via compensation or restitution). As a result, it can be difficult to disentangle (short-lived) embodiment effects from (longer lasting) neuroplasticity. Therefore, testing the direct effects of changes in the motor periphery (in our case, constraining motor degrees of freedom via the restriction of arm posture) in individuals with no neurological or peripheral deficits is necessary to directly test this issue.

As an aside, it is unclear whether the motor system is causally involved in language (this was dubbed the “necessity question” by van Elk et al., 2010). Authors such as Pulvermüller and colleagues (e.g., Pulvermüller and Fadiga, 2010) have defended the claim that the motor system plays an active role in language comprehension. However, according to others (e.g., Mahon and Caramazza, 2008), observed motor activity (neural or behavioral) might simply be an epiphenomenon, reflecting activation in sensorimotor systems following full-blown language processing. Our aim in this paper is not to provide evidence for or against either perspective. Rather, we aim to utilize a behavioral paradigm that has rarely been used and that might shed light on the question of whether, and to what extent, the state of the motor system influences cognition.

We conducted three experiments. In all experiments, participants judged as quickly and accurately as possible whether a verb and a picture of an object were compatible or incompatible. Since participants had to select one of two responses (depending on judged compatibility) this task constitutes a choice reaction time task (CRT). For the purpose of a more informative description of the task, this task was referred to as a compatibility judgment task. We tested whether constraining arm posture, resulting in a temporary elimination of afforded manual actions, would lead to delayed judgment. To test this hypothesis, two types of verbs were used: manual action (MA) verbs (i.e., verbs referring to actions performed on an object by a human hand) and non-manual action (non-MA) verbs (verbs that do not involve manipulation). We hypothesized that constrained arm posture would affect only the information processing of the MA verbs, relative to the non-MA verbs. The differences between the three experiments involved the response method (finger movements in Experiments 1 and 3 and verbal utterances in Experiment 2) and the order of presenting the object–verb pairs (an object was presented first in Experiments 1 and 2, whereas a verb was presented first in Experiment 3). To confirm that delayed responses would not be caused simply by motor factors of body constraints (e.g., difficulty or discomfort when pushing the button), we also examined the effect of constraining the arm in a task where no stimulus classification had to take place. Participants simply had to push one response button as quickly as possible, in response to the onset of the visual stimulus. Since no stimulus or response selection had to take place, this task constitutes a simple reaction time task (SRT), and in the present paper we refer to it as a word detection task.

## General methods

### Visual stimuli

**Figure 1:**
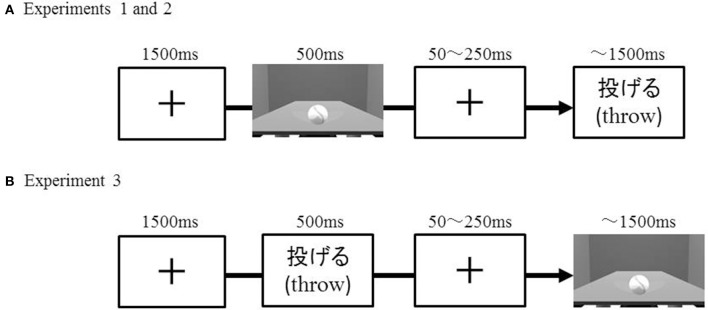
**Example of experiment stimuli (this trial represents a compatible combination) presented in Experiments 1 and 2 (A)** and Experiment 3 **(B)**.

Each trial involved the sequential presentation of a 3D object image and a verb (see Figure 1). The 3D object images were created using 3D-modeling software (MAYA, Autodesk, USA). This software allows one to create real-world scaled images. Each image depicted a table with an object placed on top of it. The dimensions and location of each object relative to those of the table were determined as if the object were within the participants' peripersonal space (approximately 30 cm). Ten objects that were manipulable with a single hand were selected (see Table 1, left column).

**Table 1:** **Combinations of words and objects**.

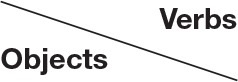	**Manual action**	**Manual action (incompatible)**	**Non-manual action**	**Non-manual action (incompatible)**
Ball	投げる (throw)	そそぐ (pour)	飛ぶ (fly)	折れる (snap)
Bottle	そそぐ (pour)	焼く (cook)	割れる (break^a^)	焦げる (burn)
Can	持つ (hold)	掘る (dig)	倒れる (fall)	鳴る (sound)
Hammer	たたく (hit)	書く (write)	落ちる (fall)	焦げる (burn)
Knife	切る (cut)	たたく (hit)	刺さる (stick)	回る (twist)
Mug cup	飲む (drink)	刺す (stab)	割れる (break^a^)	飛ぶ (fly)
Frying pan	焼く (cook)	投げる (throw)	焦げる (burn)	刺さる (stick)
Pen	書く (write)	飲む (drink)	落ちる (fall)	ぶつかる (clash)
Mobile phone	持つ (hold)	飲む (drink)	鳴る (sound)	刺さる (stick)
Tambourine	たたく (hit)	そそぐ (pour)	鳴る (sound)	滑る (slip)

a *In English “Break” can be both transitive/intransitive verbs. However, in Japanese different verbs are used as a transitive verb (割る, “waru”) and as an intransitive verb (割れる, “wareru”). Therefore, Japanese participants surely recognize that the word “割れる(wareru)” is an intransitive verb*.

*The objects are all typically operated with a single hand. English translations of the verbs re written underneath each kanji*.

We used 40 unique Japanese verbs, falling into four different categories according to verb type (MA and non-MA) and compatibility (compatible and incompatible, see Table 1). MA verbs were all transitive verbs, whereas non-MA verbs were all intransitive verbs. As an example, the stimulus-verb pair of “knife” and “cut” (MA verb) was considered compatible. The compatibility of a verb with each object had been determined on the basis of a preliminary study using a questionnaire. In this preliminary study, we preselected (a) 18 verbs relating to manual action, and are apparently compatible with at least one of the 10 objects and (b) 18 verbs which relates to non-manual action and are apparently compatible with at least one of the 10 objects. For each of 10 objects, 13 Japanese speakers were asked to select all manual action and non-manual action verbs that they considered to be compatible. Based on the results of the preliminary study, verbs that were regarded as compatible with a respective object by all participants (or 12 of 13 participants for the *hammer–fall* combination) were used as compatible verb–object pairs. In contrast, verbs that none of the participants regarded as compatible with a respective object were used as incompatible verb-object pairs.

Notably, some of object–verb pairs were based on the relationship between an instrumental object and the verb which is suitable for expressing the use of the instrumental object. We selected some object–verb pairs based on such relationship because we noticed that it was much easier for participants to judge compatibility.

### Task, apparatus, and procedure

Two experimental tasks were performed using Presentation 17.1 (Neurobehavioral Systems, Inc., USA). The main experimental task was a compatibility judgment task. Participants sat in front of a computer monitor (512 × 289 mm; FS2333, EIZO, Japan) at a distance of 40 cm from the monitor and gave compatibility judgments (see below). The apparatus for measuring response time differed between experiments. In Experiments 1 and 3, in which a response by the index finger was requested, a response box with four buttons (4 Button Curve Right, Current Designs, Inc., USA) was used. In Experiment 2 in which a vocal response was requested, a voice key (SV-1, Cedrus, USA) was used.

**Figure 2:**
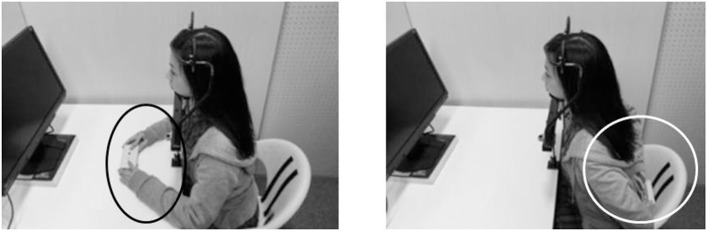
**The two hand position conditions. Left panel:** normal position, **Right panel**: constrained position.

The details of the sequential presentation in each trial are as follows (see Figure 1). A cross mark (font size 80) appeared for 1500 ms (used as a fixation point). An image of a 3D object was then presented for 500 ms. As soon as the object image disappeared, a cross mark appeared again for 50–250 ms, followed by the presentation of a verb. The verb disappeared either when participants responded or when the duration of no response exceeded 1500 ms. Participants were asked to judge as quickly as possible whether the combination of object and verb was compatible or incompatible. Participants performed this task under two hand-position conditions: normal and constrained (see Figure 2). Under the normal condition, participants put both hands in front of them on a table and operated the response buttons with both index fingers. Under the constrained condition, we asked participants to push buttons that were attached to the back support of a chair. This arrangement thus constrained participant's hand positions and arm postures. Which of the two buttons had to be pushed for a compatible pair was counterbalanced: when participants determined that the object and the verb were compatible, half of the participants were asked to lift the left finger, while the other half were asked to lift the right finger.

The other task was a word detection task. This task was necessary to determine whether the constrained posture *per se* would affect response times (i.e., the response could be delayed due to the motor factor). The same stimuli as in the compatibility judgment task were used. Participants were asked to react as quickly as possible when the verb was presented.

Each of the three experiments consisted of three parts: a preliminary check of the compatibility between the objects and verbs, the compatibility judgment task, and the word detection task. All participants performed these tasks in the same order. The compatibility judgment task was performed before the word detection task to avoid the possibility that exposure to the stimuli in the word detection task could affect response times in the compatibility judgment task. The preliminary check was necessary to confirm that all words selected as compatible were also judged to be compatible by the participants in each experiment. The results of this preliminary study showed that one or two words were not classified as compatible by one or two participants (*fall* for *can* and *hammer* in Exp. 1, *fall* for *hammer* in Exp. 2, and *fall* for *hammer* in Exp. 3). In such cases, participants were asked to treat these words as compatible.

The number of trials for the compatibility judgment task was 400: five trials for 10 objects × four verbs × two hand positions. The number of trials for the word detection task was 240: three trials for 10 objects × four verbs × two hand positions. The reason for setting a smaller number of trials for the word detection task was as follows. The word detection task was intended purely to establish a baseline of motor performance, regardless of response selection and regardless of stimulus processing. It served only to highlight the fact that, even with the hands being located behind the back, responses are equally fast. We used a smaller number of trials for the word detection task than for the compatibility judgment task in the expectation of a smaller variability for the word detection task. This was also helpful to reduce the risk that performance of the word detection task was affected by fatigue (the word detection task was always performed after the compatibility judgment task).

For both tasks, the trials were divided into two blocks depending on the hand-position condition. The order in which the hand-position condition was tested was counterbalanced among the participants. A rest period of about 3 min was scheduled within each block.

### Data analysis

The dependent variables for the compatibility judgment task were the mean reaction time and error rate; for the word detection task only the mean reaction time was calculated. We performed a three-way repeated measures analysis of variance (ANOVA) with the following factors: (a) object–verb compatibility (compatible or incompatible), (b) verb type (manual action or non-manual action), and (c) hand position (unconstrained or constrained). In addition, to investigate whether constrained arm posture would affect only the CRT obtained in the compatibility judgment task, we also performed a two-way, task type (compatibility judgment task and word detection task) and hand position (constrained or unconstrained), ANOVA with repeated measures on both factors. Significant main and interaction effects were analyzed further using Bonferroni-corrected pairwise comparisons.

## Experiment 1

We examined whether constrained arm posture would lead to the delayed judgment of verbs referring to arm actions in Experiment 1. Based on previous findings, we predicted that delayed judgment by constrained arm posture would be observed particularly for MA verbs but not for non-MA verbs because only MA verbs are related to manual actions.

### Method

Eighteen right-handed, young Japanese individuals participated (nine females and nine males, mean age = 27.5, *SD* = 6.3). Written, informed consent was obtained from each participant prior to the experiment. The protocol was approved by the ethics committee at the Tokyo Metropolitan University in accordance with the Declaration of Helsinki (authorization number H27-15). Responses were made by lifting the left or right index finger. The buttons located at the right and left edges of the response box, which contained four buttons, were used for measuring the response. Each trial began with the participant resting the right and left index finger on the right and left buttons, respectively. The participants determined whether the object and the verb were compatible or incompatible. Half of the participants lifted the right finger when an object–verb pair was compatible, whereas they lifted the left finger when a pair was incompatible. The other half of the participants lifted the left finger for when an object–verb pair was compatible, whereas they lifted the right finger when a pair was incompatible.

### Results

**Figure 3:**
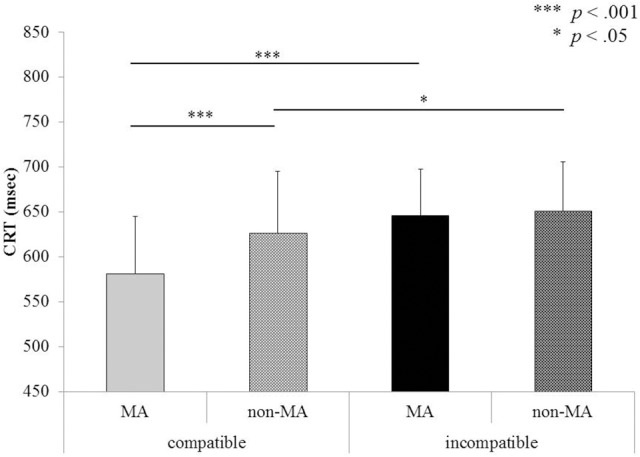
**Mean CRT for compatible and incompatible pairs, separately for the MA (manual action) and non-MA verb types in Experiment 1**.

The mean CRT (for correct responses only) under each experimental condition is shown in Table 2. The results of three-way ANOVAs for all factors are reported in Table 3. Incorrect responses (4.1% of total trials) were excluded from the statistical analysis. The main effect of hand position was significant. The CRT was significantly faster under the normal hand position than under the constrained hand position. The main effect of compatibility was significant. The CRT was significantly faster when the object–verb combination was compatible than when it was incompatible. The main effect of verb type was significant. The CRT was significantly faster for MA verbs than for non-MA verbs. Contrary to our expectation, the interaction between verb type and hand position was not significant. The interaction between compatibility and hand position was significant. Follow-up, multiple pairwise comparisons showed that the CRTs were significantly different between each pair of four conditions (i.e., two compatibilities × hand positions). The interaction between compatibility and verb type was also significant (see Figure 3). Because the most interesting contrast was observed between the compatible and incompatible combinations, multiple pairwise corrections with Bonferroni corrections were made only to statistically test the contrast. When the object–verb combination was compatible, the CRT was significantly faster for MA verbs than for the non-MA verbs.

**Table 2:** **Mean choice reaction time (CRT), Error rate, and simple reaction time (SRT) in Experiment 1 ~ 3**.

**Verb-Object Compatibility**	**Compatible**				**Incompatible**			
**Verb type**	**MA**		**Non-MA**		**MA**		**Non-MA**	
**Hand position**	**Unconstrained**	**Constrained**	**Unconstrained**	**Constrained**	**Unconstrained**	**Constrained**	**Unconstrained**	**Constrained**
**EXP. 1**								
CRT (ms)	565 ± 63	596 ± 64	604 ± 55	648 ± 75	636 ± 52	656 ± 51	639 ± 52	662 ± 58
Error rate (%)	3.1 ± 2.9	2.7 ± 2.9	4.4 ± 5.1	4.7 ± 5.1	3.8 ± 3.6	3.7 ± 2.7	6 ± 3.8	4.3 ± 3.9
SRT (ms)	323 ± 55	319 ± 41	321 ± 61	319 ± 39	328 ± 59	318 ± 41	323 ± 56	317 ± 33
**EXP. 2**								
CRT (ms)	645 ± 83	669 ± 91	686 ± 80	711 ± 87	707 ± 76	727 ± 79	707 ± 79	724 ± 83
Error rate (%)	1 ± 1.4	1.7 ± 2.7	2.7 ± 4.1	2.7 ± 3.9	1.7 ± 2.9	1.3 ± 2.1	2.7 ± 5.1	2.9 ± 4.3
SRT (ms)	379 ± 79	380 ± 85	378 ± 82	374 ± 85	381 ± 78	377 ± 83	380 ± 82	375 ± 86
**EXP. 3**								
CRT (ms)	480 ± 56	530 ± 84	516 ± 58	554 ± 102	534 ± 46	567 ± 80	556 ± 52	589 ± 87
Error rate (%)	2.9 ± 3.1	2.3 ± 2.8	5.5 ± 5.5	5.2 ± 3.5	1.8 ± 2.9	1.9 ± 2.5	2.2 ± 3.5	3.3 ± 3.7
SRT (ms)	316 ± 64	321 ± 52	316 ± 64	313 ± 55	316 ± 65	315 ± 59	315 ± 67	315 ± 55

*Standard deviations added*.

**Table 3:** **Three-way (compatibility, verb type, and hand position) analysis of variance (ANOVA) on all factors in all experiments**.

	**CRT**				**Error rate**			**SRT**		
	***Dfs***	***F***	***P***	***$\eta_{\text{p}}^{2}$***	***F***	***P***	***$\eta_{\text{p}}^{2}$***	***F***	***P***	***$\eta_{\text{p}}^{2}$***
Exp. 1 compatibility (C)	1, 17	29.58	< 0.001	0.63	0.67	0.42	0.04	0.81	0.38	0.04
verb type (V)	1, 17	44.33	< 0.001	0.72	6.12	< 0.05	0.27	1.36	0.26	0.07
hand position (H)	1, 17	16.44	< 0.001	0.49	1.16	0.29	0.06	0.37	0.55	0.02
C × V	1, 17	23.87	< 0.001	0.58	0.04	0.84	0.002	0.38	0.55	0.02
C × H	1, 17	4.55	< 0.05	0.21	1.08	0.31	0.06	2.28	0.15	0.12
V × H	1, 17	0.98	0.34	0.05	0.34	0.57	0.02	0.29	0.59	0.02
C × V × H	1, 17	0.64	0.44	0.03	2.70	0.12	0.13	0.12	0.73	0.01
Exp. 2 compatibility (C)	1, 17	29.71	< 0.001	0.63	0.01	0.92	0.001	0.16	0.69	0.01
verb type (V)	1, 17	40.16	< 0.001	0.70	5.06	< 0.05	0.23	2.45	0.14	0.12
hand position (H)	1, 17	6.87	< 0.05	0.29	0.28	0.6	0.02	0.21	0.65	0.01
C × V	1, 17	35.08	< 0.001	0.67	0.001	0.97	0.000	0.34	0.57	0.02
C × H	1, 17	2.14	0.16	0.11	0.36	0.56	0.02	0.67	0.42	0.03
V × H	1, 17	0.18	0.67	0.01	0.06	0.81	0.003	1.39	0.25	0.07
C × V × H	1, 17	0.41	0.53	0.02	0.88	0.36	0.04	0.23	0.64	0.01
Exp.3 compatibility (C)	1, 17	21.47	< 0.001	0.56	4.05	0.06	0.19	0.66	0.43	0.04
verb type (V)	1, 17	27.99	< 0.001	0.62	17.84	< 0.001	0.51	2.55	0.13	0.13
hand position (H)	1, 17	11.01	< 0.005	0.39	0.03	0.87	0.001	0.01	0.94	0.00
C × V	1, 17	1.14	0.30	0.06	5.34	< 0.05	0.24	2.31	0.15	0.12
C × H	1, 17	2.52	0.13	0.13	1.36	0.26	0.07	0.22	0.64	0.01
V × H	1, 17	0.71	0.41	0.04	0.63	0.44	0.03	1.45	0.24	0.07
C × V × H	1, 17	1.80	0.19	0.09	0.43	0.52	0.02	1.89	0.18	0.10

The mean error rate under each experimental condition is shown in Table 2. An ANOVA for the error rate showed that only the main effect of verb type was significant (see Table 3). Significantly fewer errors were found when MA verbs were presented than when non-MA verbs were presented. Neither the main effects of compatibility and hand position nor the interaction between these three factors was significant.

The mean SRT under each experimental condition is shown in Table 2. Outliers (3 × SD ± mean) were excluded from the statistical analyses. An ANOVA for the SRT showed no significant main effects or interactions.

The results of two-way ANOVA (task type × hand position) showed that the main effect of the task type was significant [*F*_(1, 17)_ = 688.0, *p* < 0.001, η_p_^2^ = 0.97]. The reaction time was significantly slower for the CRT than for the SRT. The interaction between task type and hand position was significant [*F*_(1, 17)_ = 11.66, *p* < 0.005, η_*p*_^2^ = 0.41]. When the task was a compatibility judgment, the reaction time was significantly faster under the normal hand position than under the constrained hand position. In contrast, when the task was a word detection, there was no significant difference in reaction time between the two tasks.

### Discussion

The results of the compatibility judgment task showed that, contrary to our expectation, the interaction between verb type and hand position was not significant. Instead, under the constrained condition, the CRTs became slower for both MA and non-MA verbs. We hypothesized that responding with a constrained arm posture would only affect the tasks with MA verbs because only these verbs are related to manual actions. However, the results suggest that the response was delayed regardless of whether the verbs involved manual actions. To address the reliability of the results, we investigated whether they could be replicated in the same procedure with a different response method.

Performance on the word detection task did not change even when the arm was constrained. In other words, constraining arm posture led to delayed responding only in the CRT task, but not the SRT task. This suggests that the delayed response induced by constrained arm posture in the compatibility judgment task was not merely due to the difficulty of responding via button when the arm was constrained (i.e., basic motor interference). The CRTs were slower for non-MA verbs than for MA verbs; moreover, the error rate was higher for non-MA verbs. These findings showed the difficulty in selecting the correct response for non-MA verbs.

## Experiment 2

There were two major purposes of Experiment 2. First, we investigated whether the unexpected results obtained in Experiment 1 would be replicated even with the change in response method. As stated, contrary to our expectations, constrained arm posture led to a delayed reaction time not only with MA verbs but also with non-MA verbs. We addressed whether these results were reliable. Secondly, we addressed whether the results in Experiment 1, a delay in the compatibility judgment task as a result of constrained arm posture, were not simply due to the difficulty of responding with the index fingers when the arms were constrained. For this purpose, the same tasks as those in Experiment 1 were performed with a change in the response method from a finger response to a vocal response.

### Methods

Eighteen right-handed, young Japanese individuals participated (nine females and nine males, mean age = 29.2, *SD* = 5.7). Written, informed consent was obtained from each participant prior to the experiment. The protocol was approved by the ethics committee at the Tokyo Metropolitan University in accordance with the Declaration of Helsinki (authorization number H27-15). Responses were measured from voice onset time. For the compatibility judgment task, participants were asked to say “compatible” in Japanese (“Au”) or “incompatible” in Japanese (“Awanai”). In the word detection task, half of participants responded by saying “Au” for the former half of the trials, whereas they responded with “Awanai” for the latter half of the trials.

### Results and discussion

**Figure 4:**
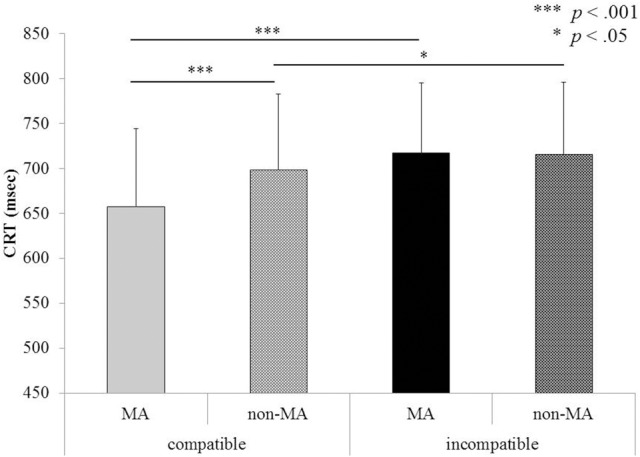
**Mean CRT for compatible and incompatible pairs, separately for the MA and non-MA verb types in Experiment 2**.

The mean CRT (for correct responses only) under each experimental condition is shown in Table 2. Incorrect responses (2.1% of total trials) were excluded from the statistical analysis. The results of three-way ANOVAs for all factors are reported in Table 3. The main effect of hand position was significant. The CRT was significantly faster under the normal hand position than under the constrained hand position. The main effect of compatibility was significant. The CRT was significantly faster when the object–verb combination was compatible than when it was incompatible. The main effect of verb type was significant. The CRT was significantly faster for MA verbs than for non-MA verbs. The interaction between compatibility and verb type was also significant (see Figure 4). Because the most interesting contrast was observed between the compatible and incompatible combinations, multiple pairwise corrections with Bonferroni corrections were made only to statistically test the contrast. When the object–verb combination was compatible, the CRT was significantly faster for MA verbs than for the non-MA verbs. The interaction between verb type and hand position was not significant.

The mean error rate under each experimental condition is shown in Table 2. An ANOVA for the error rate showed that only the main effect of verb type was significant. Significantly fewer errors were found when MA verbs were presented than when non-MA verbs were presented. Neither the main effects of compatibility and hand position nor the interaction between these three factors was significant. The mean SRT under each experimental condition is shown in Table 2. Outliers (3 × SD ± mean) were excluded from the statistical analyses. An ANOVA for the SRT showed no significant main effects or interactions.

The results of the two-way ANOVA (task type × hand position) showed that the main effect of the task type was significant [*F*_(1, 17)_ = 350.08, *p* < 0.001, η_*p*_^2^ = 0.95]. The reaction time was significantly slower for the CRT than for the SRT. The interaction between task type and hand position was significant [*F*_(1, 17)_ = 5.79, *p* < 0.05, η_*p*_^2^ = 0.25]. When the task was a compatibility judgment, the reaction time was significantly faster under the normal hand position than under the constrained hand position. In contrast, when the task was a word detection, there was no significant difference in reaction time between the two tasks.

The results of Experiment 2 mostly replicated the results of Experiment 1. Most importantly, the results of the compatibility judgment task failed to show a significant interaction between hand position and verb type. Instead, a main effect of hand position was found; under the constrained arm posture condition, the CRTs became slower for both MA and non-MA verbs. The results also replicated the findings in Experiment 1 in that (a) the compatibility judgment task was slower overall for non-MA verbs than for MA verbs; (b) the compatibility judgment task had overall slower RTs for incompatible pairs than for compatible pairs; and (c) the error rate was higher for non-MA verbs than for MA verbs. These findings showed the reliability of the findings in Experiment 1.

In Experiment 2 we changed the response method from a response by finger to a vocal response. Nevertheless, the results of Experiment 2 mostly replicated the results of Experiment 1. These findings suggest that the delayed response selection did not simply originate from the difficulty of responding with the constrained arm.

## Experiment 3

Before concluding that we did not find the evidence that restriction of arm movements only affects processing of MA verbs, an additional experiment was planned to exclude the possibility that the results, as obtained in Experiments 1 and 2, were produced merely due to the experimental condition in which a pictured stimulus of an object was presented before a verb. Gallivan et al. (2009) showed that, even when participants were asked to merely observe an object, brain areas related to motor planning and action were activated. Assuming that the same brain activity was triggered by the presentation of a pictured object stimulus in our experiments, it is possible that constrained arm posture led to delayed judgment because the information processing of a manipulable object, rather than a verb, was affected. To exclude this possibility, we conducted an experiment in which the protocol was the same as in Experiment 1, but the order of presenting the object and verb was reversed.

### Methods

Eighteen right-handed, young Japanese individuals participated (twelve females and six males, mean age = 23.9, *SD* = 5.7). Written, informed consent was obtained from each participant prior to the experiment. The protocol was approved by the ethics committee at the Tokyo Metropolitan University in accordance with the Declaration of Helsinki (authorization number H27-15). The protocol was the same as in Experiment 1, with the exception that the order of presenting an object and verb was reversed (see Figure 1B). That is, now the verb was presented before the picture, and subjects again had to judge whether the verb–picture pairs were compatible or incompatible.

### Results and discussion

The mean CRT (for correct responses only) under each experimental condition is shown in Table 2. Incorrect responses (4% of total trials) were excluded from the statistical analysis. The main effect of compatibility was significant. The CRT was significantly faster when the object–verb combination was compatible than when it was incompatible. The main effect of hand position was significant. The CRT was significantly faster under the normal hand position than under the constrained hand position. The main effect of verb type was significant. The CRT was significantly faster for the MA verb than for the non-MA verb. The interaction between verb type and hand position was not significant.

The mean error rate under each experimental condition is shown in Table 2. Significantly fewer errors were found when MA verbs were presented than when non-MA verbs were presented. A significant interaction between compatibility and verb type showed that, when the object–verb combination was compatible, the error rate was significantly lower for the MA verb than for the non-MA verb. For non-MA verbs, the error rate was significantly lower when the object–verb combination was incompatible than when it was compatible. The mean SRT under each experimental condition is shown in Table 2. Outliers (3 × SD ± mean) were excluded from the statistical analyses. An ANOVA for the SRT showed no significant main effects or interactions.

The results of two-way ANOVAs (task type × hand position) showed that the main effect of the task type was significant [*F*_(1, 17)_ = 152.23, *p* < 0.001, η_*p*_^2^ = 0.89]. The reaction time was significantly slower for the CRT than for the SRT. The main effect of hand position was significant [*F*_(1, 17)_ = 8.15, *p* < 0.05, η_*p*_^2^ = 0.32]. The reaction time was significantly faster under the normal hand position than under the constrained hand position. The interaction between task type and hand position was significant [*F*_(1, 17)_ = 9.11, *p* < 0.01, η_*p*_^2^ = 0.35]. When the task was a compatibility judgment, the reaction time was significantly faster under the normal hand position than under the constrained hand position. In contrast, when the task was a word detection, there was no significant difference in reaction time between the two tasks.

The results of Experiment 3 replicated the findings in Experiments 1 and 2 in that the CRTs became slower with constrained arm posture not only for MA verbs but also for non-MA verbs. With this finding, we excluded the possibility that this result was produced merely due to an experimental condition in which a pictured object stimulus was presented before a verb.

Considering that Japanese is an SOV language (i.e., subject, object, and verb within a sentence typically appear in that order), the verb and object pair was presented in a non-canonical order (i.e., a verb was presented before an object). Considering this fact, one might expect that the compatibility judgment task became slower in Experiment 3 than in Experiments 1 and 2. Interestingly, however, the CRT was faster in Experiment 3 than in Experiments 1 and 2. This suggests that the presentation of the verb and object pair in a non-canonical order did not impair judgment. The results also replicated the findings in Experiments 1 and 2 in that the error rate was higher for non-MA verbs than for MA verbs. In addition, when the object–verb combination was compatible, the non-MA verb had the highest rate of failure in Experiment 3. These findings showed the reliability of the findings in our study.

The results in Experiment 3 showed that, when the object-verb combination was compatible, the error rate was significantly lower for the MA verb than for the non-MA verb. Because this was not the case in Experiments 1 and 2, the results seemed to be related to the change in the protocol in Experiment 3 that a verb was presented before an object. Unfortunately, however, we have no reasonable explanation for why such a result was produced with this protocol.

## General discussion

The present study investigated whether constrained arm posture would result in a delay in the CRTs necessary for the processing of verbs referring to arm actions. We hypothesized that responding with a constrained arm posture would only affect task performances with MA verbs, because only these verbs are related to manual actions. However, the results obtained from three experiments showed that the response was delayed regardless of whether the verbs involved manual actions.

The results obtained from three experiments showed that (a) delayed CRTs with constrained arm posture in the compatibility judgment task were observed when participants reacted with their hands (Experiments 1 and 3) or their voice (Experiment 2); (b) constrained arm posture had no effects on SRTs; and (c) throughout all experiments, constraining arm posture induced slower responses, but only in the CRT task and not the SRT task. These results suggest that the results regarding delayed CRTs were not merely due to the difficulty of operating a response button when the arm was constrained (i.e., basic motor interference).

Constrained arm posture resulted in delayed CRTs regardless of the “manipulability” as symbolized by the verbs. This was different from our hypothesis that delayed CRTs with constrained arm posture would be observed only with MA verbs. This hypothesis was based on previous studies, demonstrating that the brain areas for action planning and execution are involved in the information processing of action-related words (Hauk et al., 2004; Tettamanti et al., 2005; Aziz-Zadeh et al., 2006; Péran et al., 2009; Raposo et al., 2009) but not likely in the processing of non-action-related verbs. It could be the case that (a subset of) the non-MA verbs was still associated with manual activities. For example, we had selected the verbs *fall* and *sound* as non-MA verbs, with the noun *can*. But one could also envisage a scenario whereby a falling can (plus the resulting sound) were caused may manual action, e.g., throwing or dropping. After we had conducted this experiment we obtained semantic ratings of the object-verb pairs used in Experiments 1–2. Fifteen young Japanese individuals participated (five females and ten males, mean age = 30.8, *SD* = 5.9, 10 of them had participated in Experiments 1–2) and they rated on a 5-point scale to what extent each object-verb pair would be considered a manual-action (MA)-related (0: not MA-related at all, 5: strongly MA-related). The mean and SD are shown as the Supplementary Data. Clearly, the results showed that our MA verbs were rated much higher as manual actions than our non-MA verbs, which suggests that our choice of stimulus material was adequate.

A second possible explanation for our findings could be that regardless of the verb type, information processing of all verbs takes place in the motor system. Yokoyama et al. (2006) indicated that the brain areas activated while participants read active verbs (e.g., *call*), passive verbs (e.g., *called*), and nouns showed comparable activation patterns. In their study, the activated brain areas included the bilateral inferior frontal cortex, occipital, the left middle, and inferior temporal cortices. Siri et al. (2008) also showed that action nouns (e.g., *the eating*) and verbs (infinitive verbs, e.g., *to eat*, and inflected verbs, e.g., *she/he eats*) are processed by a common neural system. Considering these previous findings, our results may have shown that the information processing of general verbs takes place in the motor system regardless of whether the verbs are related to action involving manipulation of an object. Because only verbal material was used in the present study, we could not exclude the possibility that all language processing is delayed. Future studies are necessary to address whether constraining arm posture would affect processing of action verbs but not of other types of verbs.

A third explanation is that there could be motor interference, e.g., a greater level of motor activity, which interferes with response choice when one out of two possible responses has to be selected. Based on the results obtained from the word detection task (i.e., measurement of the SRT), we excluded the possibility that delayed judgment was not simply due to the difficulty of responding with the hand (i.e., very basic motor interference). However, the choice reaction time task are not only cognitively but also motorically more complex than the simple reaction time task, because there are two available motor responses. Future studies are necessary to investigate the impact of more complex motor interference.

A final explanation could be that the motor system is only weakly coupled to semantic processing. The literature to date is in fact mixed. For example, in the field of Parkinson's disease it has been suggested that this motor disorder could lead to delayed processing of action verbs, based on the idea that mental simulation (and hence comprehension) of certain activities would be compromised in this patient group. However, some authors (e.g., Fernandino et al., 2013) found evidence for delayed action verb processing, whereas others (e.g., Kemmerer et al., 2013) found no such effect. Thus, embodiment effects are not consistently found in the literature and may be weak.

In our study, participants judged the compatibility of an object and a verb. Although the results obtained with the incompatible conditions were not directly relevant to answer our main theoretical question, these results were necessary for more practical reasons. The compatibility manipulation served one important purpose that is crucial to the experiment; namely, it forced participants to engage in deep semantic processing of the verb–object pair, thereby preventing shallow processing and keeping alertness to the stimuli high. The fact that incompatible pairs had slower CRTs than compatible pairs, which result was obtained consistently across all experiments, underscores that participants paid full attention to the verbs, so that embodied language effects (due to arm constraint) could potentially be elicited.

We would like to acknowledge several limitations to our study. First, we noticed substantial RT differences across the various object-verb pairs, for which we have no ready explanation. This source of variance may have obscured embodiment effects. Second, it could be that repetition effects (each object-verb pair was shown 5 times) were present, potentially masking relevant effects. Third, it could be that the temporary restraint of the arm was simply too brief to affect the motor representations. Future studies could manipulate the duration (>24 h) of limb immobilization and test whether this leads to a gradual change in action processing of verbs.

Despite these caveats, we believe our data can add to the debate as to whether peripheral body states can influence verb processing, and provide suggestions for fruitful innovative experiments.

## Author contributions

MY designed and conducted all of three experiments, and write the manuscript. JS supported writing introduction of the manuscript, particularly in terms of embodied cognition, and helped planning our experimental paradigm. TH supervised this study and direct the construction of the manuscript.

### Conflict of interest statement

The authors declare that the research was conducted in the absence of any commercial or financial relationships that could be construed as a potential conflict of interest.
