# Supplementary material for: Effect of Constrained Arm Posture on the Processing of Action Verbs
Source: Front Neurosci. 2017 Feb 10;11:57. doi: 10.3389/fnins.2017.00057 (PMC5300991; doi:10.3389/fnins.2017.00057)
Supplement: Supplementary file 1 [file Table1.DOCX]

Supplementary data

Table 1

Mean value (standard deviation in parenthesis) of the semantic rating on each object-verb pair

| Verbs  Objects | manual action | manual action  (incompatible) | non-manual action | non-manual action  (incompatible) |
| --- | --- | --- | --- | --- |
| Ball | throw  3.93±0.26 | pour  0.2±0.77 | fly  0.53±1.19 | snap  0.07±0.26 |
| Bottle | pour  3.6±0.83 | cook  0.2±0.77 | break  0.33±0.72 | burn  0±0 |
| Can | hold  3.93±0.26 | dig  0.47±0.83 | fall  0.27±0.7 | sound  0.33±0.72 |
| Hammer | hit  3.8±0.56 | write  0.2±0.77 | fall  0.33±1.05 | burn  0±0 |
| Knife | cut  3.73±0.59 | hit  1.33±1.18 | stick  0.53±1.13 | twist  0.2±0.77 |
| Mug cup | drink  3.27±1.28 | stab  0.2±0.77 | break  0.4±1.12 | fly  0±0 |
| Flying pan | cook  3.2±1.26 | throw  1.33±1.23 | burn  0.4±1.12 | stick  0±0 |
| Pen | write  3.93±0.26 | drink  0.33±0.9 | fall  0.27±0.7 | clash  0.2±0.77 |
| Mobile phone | hold  4±0 | drink  0.2±0.77 | sound  0.53±1.19 | stick  0.13±0.52 |
| Tambourine | hit  3.93±0.26 | pour  0.33±0.9 | sound  0.53±1.19 | slip  0.13±0.35 |
